# Knowledge, Attitude, and Practices (KAP) Regarding Diabetic Foot Care in Al-Qassim, Saudi Arabia: A Cross-Sectional Study

**DOI:** 10.7759/cureus.81475

**Published:** 2025-03-30

**Authors:** Reem Alsalamah, Saleh Hussain Alshaiban, Rayan A Alsagri, Khalil N Al-Shakhaly, Khaled Asiri, Heba Mohammad, Lajeen Alnowaisser

**Affiliations:** 1 Surgery, College of Medicine, Qassim University, Buraydah, SAU; 2 Surgery, College of Medicine, Najran University, Najran, SAU; 3 Medicine, College of Medicine, Qassim University, Buraydah, SAU; 4 Medicine, Vision College - Riyadh, Riyadh, SAU; 5 General Surgery, King Khalid University, Abha, SAU; 6 Surgery, Taibah University, Madinah, SAU; 7 Faculty of Medicine, Almaarefa College of Medicine, Riyadh, SAU

**Keywords:** al-qassim, attitude, diabetic patients, foot care, knowledge, practices, saudi arabia

## Abstract

Introduction: Diabetic foot care involves measures and practices aimed at maintaining foot health in individuals with diabetes and reducing the risk of severe, life-threatening complications. It is essential for diabetic patients to have the knowledge, attitudes, and skills necessary to enhance proper foot care, which can lead to better health outcomes and an improved quality of life.

Methods: This cross-sectional study utilized data from a sample of 647 diabetic patients residing in the Al-Qassim region of the Kingdom of Saudi Arabia. Participants completed self-administered online questionnaires, ensuring their anonymity.

Results: An overwhelming majority (604, 93.4%) of the patients had a positive attitude towards diabetic foot care, compared to only 43 (6.6%) with a poor attitude. Patients’ age and duration of diabetes were significantly associated with their attitudes towards foot care (p=0.011*; p=0.005*), with those having a family history of diabetes showing a significantly more positive attitude towards diabetic foot care. Furthermore, nearly three-quarters (480, 74.2%) of diabetic patients had good knowledge about foot care, while about one-quarter (167, 25.8%) had poor knowledge. There was no statistically significant association between knowledge regarding diabetic foot care and patients' socio-demographic attributes.

Conclusion: The study revealed a high percentage of surveyed patients with good knowledge and a positive attitude towards diabetic foot care. However, significant gaps in diabetic education were evident, as a substantial proportion of patients had not received formal education on the subject. This highlights the need for educational programs aimed at improving both knowledge and attitudes, as well as promoting good routine practices by consistently reminding patients about health practices and the risks associated with diabetes-related complications.

## Introduction

Diabetes mellitus (DM) is a very common chronic disease. In 2010, the global prevalence of adults aged 20-79 years was estimated to be 6.4%, or roughly 285 million adults. By 2030, it is expected to rise to 7.7% or roughly 439 million adults [1,2]. Globally, DM is a public health concern as a long-term, progressive metabolic disease characterized by hyperglycemia, which is brought on by an inability to metabolize insulin or by resistance to it [3-5]. According to data from the World Health Organization (WHO), Saudi Arabia has the second-highest diabetes prevalence in the Middle East and the seventh-highest worldwide [6].

Every day, more and more diabetic patients experience complications related to their feet and lower limbs; globally, this affects between 50 and 60 million people [7]. One of the complications of diabetes that results from peripheral arterial disease (PAD) is diabetic foot, which is characterized by sensory neuropathy in the feet of diabetic patients [8,9]. Men are more likely than women to develop diabetic foot complications, and type 2 diabetics are more likely than type 1 diabetics to do so [10]. With an annual foot ulceration rate of roughly 2%, lower-middle-income economies are more likely than high-income ones to experience foot ulcerations [7,11]. Complications from diabetic feet are a major cause of morbidity, low quality of life, and early death [7,12]. The main risk factors for diabetic foot disease are uncontrolled hyperglycemia, the length of diabetes, peripheral artery disease, peripheral neuropathy, foot deformity, recurrent minor traumas, and infections [7,13-17]. Furthermore, less than one-third of practitioners recognize the signs of diabetic peripheral neuropathy. Unusually high morbidity and mortality are caused by the misdiagnosis that follows [18].

Maintaining adequate care of the feet has always been crucial to reducing the risk of diabetic foot problems and their consequences. With appropriate foot care practices, such as routine foot washing and drying, routine foot assessments, proper nail care, and appropriate footwear, diabetic foot and its complications can be avoided or identified early. Patients should take self-foot care seriously and incorporate it into their daily routine [7,19].

To the best of our knowledge, no research has been done to assess diabetic patients' awareness of diabetic foot care in Al-Qassim province, Saudi Arabia. Nevertheless, some studies have been conducted in Saudi Arabia to determine diabetic patients' awareness of diabetic foot care. Therefore, this study aims to assess the attitudes and knowledge of diabetic patients in the province of Al-Qassim regarding diabetic foot care.

Objectives

The study aims to assess the attitudes and knowledge of diabetic patients in the province of Al-Qassim regarding diabetic foot care.

## Materials and methods

Study design

This study employed a descriptive, questionnaire-based, cross-sectional design. The research was conducted online among individuals residing in the Al-Qassim region of Saudi Arabia. The study duration extended from May 2024 to October 2024.

Study population

The target population comprised individuals residing in the region, as per the latest statistics from the General Authority for Statistics in Saudi Arabia, which reported a population of 1,488,285 [20].

Sample size

The sample size was determined using the Raosoft Sample Size Calculator [21], with a margin of error of 5% and a confidence level of 95%. Based on these parameters, the estimated sample size was 377 participants. A convenience sampling technique was utilized to recruit participants.

Inclusion and exclusion criteria

The inclusion and exclusion criteria for participant eligibility are summarized in Table 1.

**Table 1:** Inclusion and Exclusion Criteria

Criteria	Details
Inclusion Criteria	Anyone in the Al-Qassim region of Saudi Arabia aged above 18
	Anyone known to have type 1 or type 2 diabetes, whether Saudi or non-Saudi
Exclusion Criteria	Anyone who has not accepted to participate

Data collection 

Data were collected using an online, self-administered questionnaire distributed via Google Forms (Appendix 1). The questionnaire was disseminated across multiple social media platforms, including WhatsApp, Twitter, and Instagram. Prior to participation, individuals were required to provide electronic informed consent. The research team ensured the accessibility of the questionnaire and provided assistance to participants as needed.

Data management and analysis plan

All participants who agreed to participate were provided with an informed consent form. The online questionnaire was designed to prevent incomplete submissions by requiring responses to all items. Upon collection, the data were reviewed to ensure completeness and accuracy. Responses were exported to Microsoft Excel (Redmond, WA, USA) for encoding and confidentiality maintenance. No personally identifiable information, such as names or contact details, was collected. The data were securely stored with password protection, accessible only to the research team. For statistical analysis, the data were transferred to SPSS (IBM Corp., Armonk, NY, USA). Continuous variables, such as age, were reported as mean ± standard deviation, while categorical variables, such as gender, were described using frequencies and percentages. The chi-square test was employed to compare categorical variables, with a p-value of less than 0.05 considered statistically significant.

Ethical considerations

Ethical considerations were rigorously adhered to throughout the study. Participant confidentiality and data privacy were prioritized, and no identifying information was collected. Ethical approval for the study was obtained from the ethical committee of Qassim University's medical college (approval number: CL-20240516-11).

Knowledge classification

To assess participants' knowledge about foot care, 12 key questions were included in the questionnaire. Each correct or appropriate response indicating accurate knowledge was awarded one point, while incorrect or "unsure" responses received zero points, resulting in a maximum possible score of 12. Based on the total score, participants were classified into two categories: those who scored 8 or more points (≥66.7%) were considered to have good knowledge, while those who scored 7 or fewer points (<66.7%) were categorized as having poor knowledge.

## Results

Table 2 shows that a total of 647 diabetic patients completed the questionnaires. Among them, more than half (356, 55.0%) were male, and nearly one-third (194, 30.0%) were aged between 18 and 29 years. The vast majority of participants were Saudis (607, 93.8%), with a notable proportion being married (399, 61.7%). Most had attained a university education or higher (478, 73.9%). In terms of employment, nearly one-third (209, 32.3%) were government sector employees, and about one-quarter (164, 25.3%) reported a monthly income of over 15,000 riyals. Additionally, a significant number of patients had a family history of diabetes (406, 62.8%), while a considerable proportion (245, 37.9%) reported having fewer than two clinical visits per year.

**Table 2:** Socio-demographic data of the participants (N=647) Socio-demographic information presented in frequencies (n) and proportion (%)

Variables	Category	Frequency and percentage n (%)
Sex	Female	291 (45.0%)
	Male	356 (55.0%)
Age in years	Under 18 years	17 (2.6%)
	18-29	194 (30.0%)
	30-39	101 (15.6%)
	40-49	142 (21.9%)
	50-59	118 (18.2%)
	60 years and above	75 (11.6%)
Nationality	Saudi	607 (93.8%)
	Non-Saudi	40 (6.2%)
Marital status	Single	223 (34.5%)
	Married	399 (61.7%)
	Divorced	12 (1.9%)
	Widow	13 (2.0%)
Education level	Intermediate education	29 (4.5%)
	Primary education and below	14 (2.2%)
	Secondary education	126 (19.5%)
	University education or higher	478 (73.9%)
Profession	Government sector employee	209 (32.3%)
	Private sector employee	95 (14.7%)
	Retired	116 (17.9%)
	Student	132 (20.4%)
	Unemployed	95 (14.7%)
Monthly income in Riyals	Less than 5000 riyals	240 (37.1%)
	5000-10000 riyals	132 (20.4%)
	10000-15000 riyals	111 (17.2%)
	More than 15000 riyals	164 (25.3%)
Duration of diabetes in years	Less than a year	27 (4.2%)
	1-5 years	65 (10.0%)
	6-10 years	52 (8.0%)
	More than 10 years	97 (15.0%)
	Family history	406 (62.8%)
Number of clinic visits per year	Less than two visits	245 (37.9%)
	2-4 visits	229 (35.4%)
	5-7 visits	102 (15.8%)
	More than 7 visits	71 (11.0%)

Table 3 shows the patients’ knowledge regarding diabetic foot health. The overwhelming majority of patients (590, 91.2%) were aware that diabetics are more likely to develop gangrene in the foot. Additionally, 577 patients (91.2%) recognized that regular foot care can prevent many diabetic foot problems, and 575 patients (88.9%) understood that diabetics may lose sensation in their feet. Furthermore, 574 patients (88.7%) knew that loss of sensation can make diabetics more vulnerable to foot ulcers.

**Table 3:** Knowledge measurement Knowledge attributes presented in frequencies (n) and proportion (%)

Attributes	Categories	n (%)
Diabetics may experience reduced blood flow to their feet	Strongly agree	345 (53.3%)
	I agree	211 (32.6%)
	Neutral	47 (7.3%)
	I do not agree	39 (6.0%)
	Strongly disagree	5 (0.8%)
Am aware that diabetics may lose sensation in their feet	Strongly agree	373 (57.7%)
	I agree	202 (31.2%)
	Neutral	39 (6.0%)
	I do not agree	27 (4.2%)
	Strongly disagree	6 (0.9%)
I understand that diabetes can get foot ulcers	Strongly agree	401 (62.0%)
	I agree	194 (30.0%)
	Neutral	29 (4.5%)
	I do not agree	18 (2.8%)
	Strongly disagree	5 (0.8%)
Diabetics are more likely to develop gangrene in the foot	Strongly agree	430 (66.5%)
	I agree	160 (24.7%)
	Neutral	37 (5.7%)
	I do not agree	15 (2.3%)
	Strongly disagree	5 (0.8%)
Loss of sensation in the feet can make diabetics vulnerable to foot ulcers	Strongly agree	381 (58.9%)
	I agree	193 (29.8%)
	Neutral	41 (6.3%)
	I do not agree	28 (4.3%)
	Strongly disagree	4 (0.6%)
Am aware that decreased blood flow to the feet can make diabetics prone to foot ulcers	Strongly agree	377 (58.3%)
	I agree	193 (29.8%)
	Neutral	47 (7.3%)
	I do not agree	25 (3.9%)
	Strongly disagree	5 (0.8%)
I know that diabetes can cause changes in the shape of the foot	Strongly agree	323 (49.9%)
	I agree	178 (27.5%)
	Neutral	82 (12.7%)
	I do not agree	47 (7.3%)
	Strongly disagree	17 (2.6%)
I understand that high blood sugar levels can damage the blood vessels in the feet	Strongly agree	355 (54.9%)
	I agree	204 (31.5%)
	Neutral	56 (8.7%)
	I do not agree	24 (3.7%)
	Strongly disagree	8 (1.2%)
I know the signs of diabetic foot infection	Strongly agree	237 (36.6%)
	I agree	156 (24.1%)
	Neutral	133 (20.6%)
	I do not agree	91 (14.1%)
	Strongly disagree	30 (4.6%)
I understand the importance of wearing proper shoes for people with diabetes	Strongly agree	360 (55.6%)
	I agree	198 (30.6%)
	Neutral	52 (8.0%)
	I do not agree	28 (4.3%)
	Strongly disagree	9 (1.4%)
I know that smoking can increase foot complications in diabetics	Strongly agree	345 (53.3%)
	I agree	174 (26.9%)
	Neutral	76 (11.7%)
	I do not agree	41 (6.3%)
	Strongly disagree	11 (1.7%)
I know that regular foot care can prevent many diabetic foot problems	Strongly agree	405 (62.6%)
	I agree	172 (26.6%)
	Neutral	40 (6.2%)
	I do not agree	23 (3.6%)
	Strongly disagree	7 (1.1%)

Table 4 presents the results of the patients’ attitudes toward diabetic foot care. The vast majority of patients (615, 95.1%) recognized nutrition as an important factor in controlling blood sugar levels. Most participants agreed that exercise helps maintain diabetic foot health (566, 87.5%) and that regular visit to a podiatrist are necessary for diabetics (550, 85.0%). Additionally, 534 patients (82.5%) expressed a willingness to use special shoes to protect their feet. Interestingly, nearly half of the patients (327, 50.5%) had received education about diabetic foot care from a healthcare provider.

**Table 4:** Attitude measurement Attitude measurement attributes presented in frequencies (n) and proportion (%)

Attributes	Categories	n (%)
Nutrition is an important factor in controlling blood sugar levels	Yes	615 (95.1%)
	No	13 (2.0%)
	Maybe	19 (2.9%)
Am willing to use special shoes to protect my feet	Yes	534 (82.5%)
	No	35 (5.4%)
	Maybe	78 (12.1%)
I think regular visits to a podiatrist are necessary for diabetics	Yes	550 (85.0%)
	No	29 (4.5%)
	Maybe	68 (10.5%)
I believe exercise helps maintain diabetic foot health	Yes	566 (87.5%)
	No	28 (4.3%)
	Maybe	53 (8.2%)
I have received education about diabetic foot care from a health care provider	Yes	327 (50.5%)
	No	231 (35.7%)
	Maybe	89 (13.8%)

Table 5 illustrates the patients’ practices regarding diabetic foot care. Among the 188 patients with sores or wounds (29.1%), more than half (108, 57.4%) sought medical attention for treatment. Most patients reported washing their feet daily (562, 86.9%) and cutting their nails straight across while filing the edges (502, 77.6%), regardless of whether they had diabetes. Furthermore, a considerable proportion refrained from using harsh chemicals or over-the-counter treatments on their feet without consulting a doctor (443, 68.5%). Surprisingly, less than half of the patients reported wearing socks designed specifically for people with diabetes (197, 30.4%), while only 204 (31.5%) of them self-inspected their feet daily.

**Table 5:** Practice measurement Practice measurement presented in frequencies (n) and proportion (%)

Attributes	Categories	n (%)
Have you ever had sores or wounds on your feet?	Yes	188 (29.1%)
	No	459 (70.9%)
If yes, in the question above, how did you deal with them?	I treated it myself	80 (42.6%)
	I sought medical attention	108 (57.4%)
I check my feet daily, whether I have diabetes or not	Yes	278 (43.0%)
	No	214 (33.1%)
	Sometimes	155 (24.0%)
I wash my feet daily, whether I have diabetes or not	Yes	562 (86.9%)
	No	42 (6.5%)
	Sometimes	43 (6.6%)
I dry my feet between toes after washing, whether I have diabetes of not	Yes	344 (53.2%)
	No	181 (28.0%)
	Sometimes	122 (18.9%)
I use lotion/moisturizer on my feet, whether I have diabetes or not	Yes	334 (51.6%)
	No	169 (26.1%)
	Sometimes	144 (22.3%)
I avoid walking barefoot, whether I have diabetes or not	Yes	424 (65.5%)
	No	128 (19.8%)
	Sometimes	95 (14.7%)
I check my shoes before wearing them, whether I have diabetes or not	Yes	329 (50.9%)
	No	203 (31.4%)
	Sometimes	115 (17.8%)
I protect my feet and keep them away from high and low temperatures, whether I have diabetes of not	Yes	420 (64.9%)
	No	128 (19.8%)
	Sometimes	99 (15.3%)
I cut my nails straight across and file the edges, whether I have diabetes or not	Yes	502 (77.6%)
	No	73 (11.3%)
	Sometimes	72 (11.1%)
I wear socks designed specifically for people with diabetes, whether I have diabetes or not	Yes	197 (30.4%)
	No	359 (55.5%)
	Sometimes	91 (14.1%)
I use a mirror to check the soles of my feet if I have difficult seeing them, whether I have diabetes or not	Yes	204 (31.5%)
	No	358 (55.3%)
	Sometimes	85 (13.1%)
I avoid using heating pads or hot water bottles on my feet, whether I have diabetes or not	Yes	315 (48.7%)
	No	254 (39.3%)
	Sometimes	78 (12.1%)
I refrain from using harsh chemicals or over-the-counter treatment on my feet without consulting a doctor	Yes	443 (68.5%)
	No	155 (24.0%)
	Sometimes	49 (7.6%)
I monitor my blood sugar levels regularly	Yes	376 (58.1%)
	No	166 (25.7%)
	Sometimes	105 (16.2%)
I do exercise regularly to help control your blood sugar levels	Yes	325 (50.2%)
	No	151 (23.3%)
	Sometimes	171 (26.4%)

Table 6 shows the patients’ knowledge measurement levels based on the questions provided in the questionnaire. The knowledge measurement consisted of 12 questions categorized into five subscales: strongly agree, I agree, neutral, I do not agree and strongly disagree. The response options were as follows: 5=strongly agree; 4=I agree; 3=neutral; 2=I do not agree; 1=strongly agree. The highest score any participant could get was 60, and the lowest score any respondent could get was 12. Data was converted to composite scores. The scoring system was categorized as follows: Good knowledge (48-60) and poor knowledge (12-47). Data was then analyzed to find out the levels of knowledge among the diabetic patients.

**Table 6:** Association between socio-demographic data and the patients’ level of knowledge regarding diabetic foot care * Significant at p<0.05 level.

Socio-demographic variables		Knowledge level		Cramer’ V, DF	P-value
		Poor	Good		
Sex	Female	80 (27.5%)	211 (72.5%)	0.035, 1	0.377
	Male	87 (24.4%)	269 (75.6%)		
Age in years	Under 18 years	6 (35.3%)	11 (64.7%)	0.113, 5	0.140
	18-29	56 (28.9%)	138 (71.1%)		
	30-39	33 (32.7%)	68 (67.3%)		
	40-49	27 (19.0%)	115 (81.0%)		
	50-59	28 (23.7%)	90 (76.3%)		
	60 years and above	17 (22.7%)	58 (77.3%)		
Nationality	Saudi	160 (26.4%)	447 (73.6%)	0.049, 1	0.215
	Non-Saudi	7 (17.5%)	33 (82.5%)		
Marital status	Single	62 (27.8%)	161 (72.2%)	0.095, 3	0.120
	Married	100 (25.1%)	299 (74.9%)		
	Divorced	0 (0.0%)	12 (100.0%)		
	Widow	5 (38.5%)	8 (61.5%)		
Education level	Intermediate education	4 (13.8%)	25 (86.2%)	0.092, 3	0.138
	Primary education and below	6 (42.9%)	8 (57.1%)		
	Secondary education	28 (22.2%)	98 (77.8%)		
	University education or higher	129 (27.0%)	349 (73.0%)		
Profession	Government sector employee	40 (19.1%)	169 (80.9%)	0.109, 4	0.105
	Private sector employee	27 (28.4%)	68 (71.6%)		
	Retired	32 (27.6%)	84 (72.4%)		
	Student	38 (28.8%)	94 (71.2%)		
	Unemployed	30 (31.6%)	65 (68.4%)		
Monthly income in Riyals	Less than 5000 riyals	68 (28.3%)	172 (71.7%)	0.064, 3	0.444
	5000-10000 riyals	36 (27.3%)	96 (72.7%)		
	10000-15000 riyals	28 (25.2%)	83 (74.8%)		
	More than 15000 riyal	35 (21.3%)	129 (78.7%)		
Duration of diabetes in years	Less than a year	10 (37.0%)	17 (63.0%)	0.109, 4	0.105
	1-5 years	24 (36.9%)	41 (63.1%)		
	6-10 years	13 (25.0%)	39 (75.0%)		
	More than 10 years	20 (20.6%)	77 (79.4%)		
	Family history	100 (24.6%)	306 (75.4%)		
Number of clinic visits per year	Less than two visits	68 (27.8%)	177 (72.2%)	0.050, 3	0.661
	2-4 visits	60 (26.2%)	169 (73.8%)		
	5-7 visits	24 (23.5%)	78 (76.5%)		
	More than 7 visits	15 (21.1%)	56 (78.9%)		

Table 6 presents the association between patients’ socio-demographic data and their levels of knowledge regarding diabetic foot care. The study revealed that nearly three-quarters (480, 74.2%) of the diabetic patients had good knowledge, while about one-quarter (167, 25.8%) of them had poor knowledge regarding diabetic foot care. The study found no significant association between patients’ knowledge levels about diabetic foot care and the social demographic variables (p>0.005).

Table 7 presents the association between patients’ socio-demographic data and their levels of attitude towards diabetic foot care. The study revealed that a significant majority (604, 93.4%) of the diabetic patients had a good attitude, while only 43 (6.6%) of them had a poor attitude towards diabetic foot care. Patients’ age and duration of diabetes were found to be significantly associated with their attitude towards diabetic foot care (p=0.011; 0.005*). However, there was no significant association between patients’ attitude towards diabetic foot care with other social demographic variables (p>0.005).

**Table 7:** Association between socio-demographic data and the patients’ attitude towards diabetic foot care * Significant at p<0.05 level.

Socio-demographic variables		Attitude level		Cramer’ V, DF	P-value
		Poor	Good		
Sex	Female	16 (5.5%)	275 (94.5%)	0.042, 1	0.289
	Male	27 (7.6%)	329 (92.4%)		
Age in years	Under 18 years	4 (23.5%)	13 (76.5%)	0.151, 5	0.011*
	18-29	19 (9.8%)	175 (90.2%)		
	30-39	4 (4.0%)	97 (96.0%)		
	40-49	5 (3.5%)	137 (96.5%)		
	50-59	6 (5.1%)	112 (94.9%)		
	60 years and above	5 (6.7%)	70 (93.3%)		
Nationality	Saudi	39 (6.4%)	568 (93.6%)	0.035, 1	0.379
	Non-Saudi	4 (10.0%)	36 (90.0%)		
Marital status	Single	20 (9.0%)	203 (91.0%)	0.071, 3	0.355
	Married	21 (5.3%)	378 (94.7%)		
	Divorced	1 (8.3%)	11 (91.7%)		
	Widow	1 (7.7%)	12 (92.3%)		
Education level	Intermediate education	1 (3.4%)	28 (96.6%)	0.054, 3	0.601
	Primary education and below	2 (14.3%)	12 (85.7%)		
	Secondary education	9 (7.1%)	117 (92.9%)		
	University education or higher	31 (6.5%)	447 (93.5%)		
Profession	Government sector employee	11 (5.3%)	198 (94.7%)	0.087, 4	0.296
	Private sector employee	6 (6.3%)	89 (93.7%)		
	Retired	8 (6.9%)	108 (93.1%)		
	Student	14 (10.6%)	118 (89.4%)		
	Unemployed	4 (4.2%)	91 (95.8%)		
Monthly income in Riyals	Less than 5000 riyals	18 (7.5%)	222 (92.5%)	0.032, 3	0.882
	5000-10000 riyals	9 (6.8%)	123 (93.2%)		
	10000-15000 riyals	7 (6.3%)	104 (93.7%)		
	More than 15000 riyal	9 (5.5%)	155 (94.5%)		
Duration of diabetes in years	Less than a year	5 (18.5%)	22 (81.5%)	0.152, 4	0.005*
	1-5 years	9 (13.8%)	56 (86.2%)		
	6-10 years	4 (7.7%)	48 (92.3%)		
	More than 10 years	7 (7.2%)	90 (92.8%)		
	Family history	18 (4.4%)	388 (95.6%)		
Number of clinic visits per year	Less than two visits	17 (6.9%)	228 (93.1%)	0.015, 3	0.985
	2-4 visits	15 (6.6%)	214 (93.4%)		
	5-7 visits	6 (5.9%)	96 (94.1%)		
	More than 7 visits	5 (7.0%)	66 (93.0%)		

## Discussion

Given the high incidence rate of diabetes in Saudi Arabia, there is a knowledge gap among diabetic patients regarding foot care, which increases the risk of serious complications. Understanding patients’ attitudes and knowledge may help tailor treatment strategies to enhance foot care behaviors, prevent complications, and improve patients’ quality of life and overall health outcomes [22]. This study aimed to assess the attitude and knowledge of diabetic patients in the province of Al-Qassim regarding diabetic foot care.

The study revealed that nearly three-quarters (480, 74.2%) of diabetic patients had good knowledge about foot care, while about one-quarter (167, 25.8%) had poor knowledge. The level of knowledge in our study was significantly higher than in other studies conducted in Saudi Arabia, where Solan et al. [23] reported good knowledge in only 53.6% of patients, and Al-Aboudi et al. [24] reported 13.3%. This wide variation in the prevalence of knowledge observed across studies in Saudi Arabia may be attributed to differences in data collection methods and variations in sample sizes. Notably, the study found that the majority of patients (590, 91.2%) were aware that diabetics are more likely to develop gangrene in the foot and that regular foot care can prevent many diabetic foot problems (577, 91.2%).

Regarding the attitude, an overwhelming majority (604, 93.4%) of the patients had a positive attitude towards diabetic foot care, compared to only 43 (6.6%) with a poor attitude. It was observed that a significant majority of patients recognized the importance of nutrition in controlling blood sugar levels (615, 95.1%) and the role of exercise in maintaining diabetic foot health (566, 87.5%). Additionally, most patients expressed a willingness to use special shoes to protect their feet (534, 82.5%).

The study revealed that patients aged 18 years and older had a significantly better attitude towards diabetic foot care compared to those under 18 years (p = 0.011). This difference in attitude levels may be attributed to the greater experience older individuals have in managing diabetes, leading to increased awareness of the importance of foot care and the risks of potential complications [25]. Furthermore, patients with a family history of diabetes had a significantly better attitude towards diabetic foot care compared to those who had lived with the disease for a shorter duration (p = 0.005). These findings align with those of a study conducted by Shah et al., which reported that patients with a long-standing history of diabetes had a better attitude towards diabetic foot care [26]. Surprisingly, about 327 patients (50.5%) had received education about diabetic foot care from a healthcare provider, highlighting significant gaps in educational uptake, as nearly half (320, 49.5%) had not received formal education on this topic. This underscores the need for awareness programs to enhance patients' knowledge and motivate them to adopt a positive attitude towards diabetic foot care.

Regarding foot care practices, more than half of the patients (108, 57.4%) with sores or wounds sought medical attention, indicating adherence to proper health practices among a substantial proportion. However, most participants did not engage in good routine practices, as less than half (204, 31.5%) self-inspected their feet daily, and only about 197 (30.4%) wore socks specifically designed for people with diabetes. While these rates are higher compared to previous studies - where only 22.2% of participants in Goie and Naidoo [27] examined their feet and only 14% in Hasnain and Sheikh [28] reported good foot care practices - there is still a need to motivate healthcare practitioners to educate diabetic patients and consistently remind them about routine health practices and the risks of diabetes-related complications.

It is crucial to consider the limitations of the study while assessing the results. The cross-sectional design could only assess the relationship between the study attributes, but not their causalities. The study findings could be generalized to the entire Saudi Arabian population given that it was conducted in only one region, Al-Qassim. Furthermore, since the study relied on self-reported online survey data, biases related to recollection and social desirability may have compromised its reliability and accuracy.

## Conclusions

The study revealed a high percentage of surveyed patients with good knowledge and a positive attitude towards diabetic foot care. However, significant gaps in diabetic education were evident, as a substantial proportion of patients had not received formal education on the subject. This highlights the need for educational programs aimed at improving both knowledge and attitudes, as well as promoting good routine practices by consistently reminding patients about health practices and the risks associated with diabetes-related complications.

## Appendices

Appendix 1

Survey on Knowledge About Foot Care for Diabetic Patients in the Qassim Region

Consent to Participate

Do you agree to participate in this survey?

• Yes • No

Demographic and Residential Information

Do you reside in the Qassim region?

• Yes • No

Personal Information

Gender:

• Male

• Female

Age:

• Under 18 years

• 18-29 years

• 30-39 years

• 40-49 years

• 50-59 years

• 60 years and above

Nationality:

• Saudi

• Non-Saudi

Marital Status:

• Married

• Single

• Divorced

• Widowed

Education Level:

• Primary education or below

• Middle school education

• High school education

• University degree or higher

Occupation:

• Government sector employee

• Private sector employee

• Unemployed

• Retired

• Student

Monthly Income:

• Less than 5,000 SAR

• 5,000-10,000 SAR

• 10,000-15,000 SAR

• More than 15,000 SAR

Duration of Diabetes Diagnosis:

• Less than a year

• 1-5 years

• 6-10 years

• More than 10 years

• A close relative has diabetes

Number of Clinic Visits Per Year:

• Less than 2 visits

• 2-4 visits

• 5-7 visits

• More than 7 visits

Knowledge Assessment

Are you aware that diabetic patients may experience reduced blood flow to their feet?

• Strongly agree • Agree • Neutral • Disagree • Strongly disagree

Do you know that diabetic patients may lose sensation in their feet?

• Strongly agree • Agree • Neutral • Disagree • Strongly disagree

Do you understand that diabetic patients can develop foot ulcers?

• Strongly agree • Agree • Neutral • Disagree • Strongly disagree

Are you aware of the possibility of diabetic patients developing gangrene in their feet?

• Strongly agree • Agree • Neutral • Disagree • Strongly disagree

Do you know that loss of sensation in the feet can make diabetic patients more prone to foot ulcers?

• Strongly agree • Agree • Neutral • Disagree • Strongly disagree

Are you aware that reduced blood flow to the feet can make diabetic patients more prone to foot ulcers?

• Strongly agree • Agree • Neutral • Disagree • Strongly disagree

Do you know that diabetes can cause changes in foot shape?

• Strongly agree • Agree • Neutral • Disagree • Strongly disagree

Are you aware that high blood sugar levels can damage blood vessels in the feet?

• Strongly agree • Agree • Neutral • Disagree • Strongly disagree

Do you know the signs of diabetic foot infection?

• Strongly agree • Agree • Neutral • Disagree • Strongly disagree

Do you understand the importance of wearing appropriate footwear for diabetic patients?

• Strongly agree • Agree • Neutral • Disagree • Strongly disagree

Are you aware that smoking can worsen foot complications in diabetic patients?

• Strongly agree • Agree • Neutral • Disagree • Strongly disagree

Do you know that regular foot care can prevent many diabetic foot problems?

• Strongly agree • Agree • Neutral • Disagree • Strongly disagree

Attitude Assessment

Do you consider nutrition an important factor in controlling blood sugar levels?

• Yes

• No

• Maybe

Would you be willing to wear special footwear to protect your feet?

• Yes

• No

• Maybe

Do you think regular visits to a podiatrist are necessary for diabetic patients?

• Yes

• No

• Maybe

Do you believe that physical exercise helps maintain diabetic foot health?

• Yes

• No

• Maybe

Have you received education about diabetic foot care from a healthcare provider?

• Yes

• No

• Maybe

Have you ever had foot ulcers or wounds? How did you handle them?

• Yes, I sought medical care

• Yes, I treated them myself

• No

Foot Care Practices

Do you check your feet daily, whether you have diabetes or not?

• Yes • No • Sometimes

Do you wash your feet daily, whether you have diabetes or not?

• Yes • No • Sometimes

Do you dry your feet and between your toes after washing, whether you have diabetes or not?

• Yes • No • Sometimes

Do you use lotion/moisturizer on your feet, whether you have diabetes or not?

• Yes • No • Sometimes

Do you avoid walking barefoot, whether you have diabetes or not?

• Yes • No • Sometimes

Do you check your shoes before wearing them, whether you have diabetes or not?

• Yes • No • Sometimes

Do you protect your feet from extreme hot and cold temperatures, whether you have diabetes or not?

• Yes • No • Sometimes

Do you trim your nails straight across and file the edges, whether you have diabetes or not?

• Yes • No • Sometimes

Do you wear socks designed for diabetic patients, whether you have diabetes or not?

• Yes • No • Sometimes

Do you use a mirror to check the soles of your feet if you have trouble seeing them, whether you have diabetes or not?

• Yes • No • Sometimes

Do you avoid using heating pads or hot water bottles on your feet, whether you have diabetes or not?

• Yes • No • Sometimes

Do you refrain from using harsh chemicals or over-the-counter treatments on your feet without consulting a doctor?

• Yes • No • Sometimes

Do you monitor your blood sugar levels regularly?

• Yes • No • Sometimes

Do you engage in regular physical activity to help control your blood sugar levels?

• Yes • No • Sometimes
